# Vacuum‐Induced Surface Reconstruction of Mn–Ce Oxides for Low‐Temperature Coupled Redox Catalysis

**DOI:** 10.1002/anie.2739215

**Published:** 2026-09-21

**Authors:** Yong Yin, Yide Jiang, Zichen Xu, Min Liu, Emiliano Cortés, Yifei Sun

**Affiliations:** ^1^ Key Laboratory of Agro‐Forestry Environmental Processes and Ecological Regulation of Hainan Province School of Environmental Science and Engineering Hainan University Haikou China; ^2^ Hainan International One Health Institute Hainan University Haikou Hainan China; ^3^ Hainan Province Key Laboratory of One Health Hainan University Haikou Hainan China; ^4^ Hunan Joint International Research Center For Carbon Dioxide Resource Utilization School of Physics Central South University Changsha Hunan China; ^5^ School of Metallurgy and Environment Central South University Changsha Hunan China; ^6^ Nanoinstitute Munich Faculty of Physics Ludwig‐Maximilians‐Universität München München Germany

**Keywords:** coupled redox reaction, electron‐rich interface, near‐surface reconstruction, oxygen‐mediated catalysis, surface MnO*_x_* enrichment

## Abstract

Low‐temperature redox catalysis over mixed oxides is typically optimized by tuning bulk composition, yet the decisive chemistry occurs at the surface and subsurface interface. Here, we show that this interface can be deliberately reconstructed to enhance Mn–Ce oxide catalysis. Sequential vacuum annealing followed by reduced‐pressure O_2_ treatment induces near‐surface Mn enrichment in an as‐prepared Mn–Ce oxide while largely preserving the fluorite CeO_2_ framework. This reconstruction increases the near‐surface abundance of Mn‐containing species, modifies the Mn–Ce–O interfacial electronic and redox environment, and facilitates oxygen activation and redox cycling. Under coupled conditions, R‐MnCe achieved 95.5% NO*_x_* conversion at 100°C and complete chlorobenzene conversion at 200°C. In situ spectroscopy and DFT calculations reveal that interfacial electron redistribution lowers the oxygen activation barrier and sustains activated oxygen species that connect NH_3_‐SCR and chlorobenzene oxidation through a shared oxygen‐mediated pathway. These results highlight near‐surface reconstruction as a promising postsynthetic strategy for improving noble‐metal‐free mixed‐oxide catalysts.

## Graphical abstract

Near‐surface reconstruction enhances low‐temperature redox catalysis in Mn–Ce mixed oxides. Sequential vacuum annealing and reduced‐pressure O_2_ treatment enrich Mn near the surface while preserving the CeO_2_ fluorite framework, promoting interfacial electron redistribution, oxygen activation, and redox cycling. The reconstructed catalyst enables NO_x_ reduction and chlorobenzene oxidation via an oxygen‐mediated pathway.

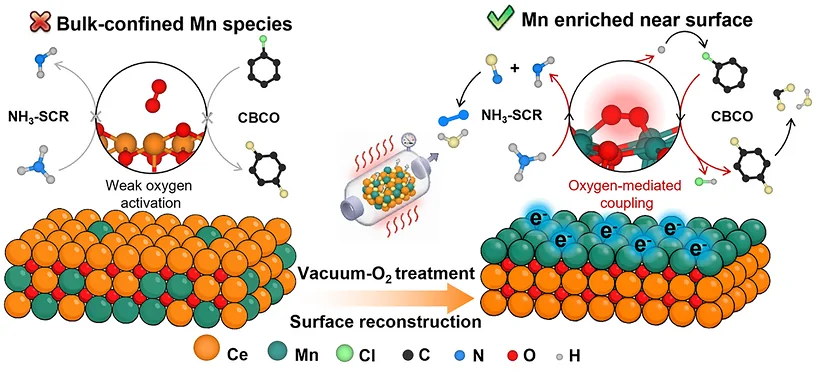

## 1 Introduction

Low‐temperature oxygen‐mediated redox catalysis is important in chemical transformation and environmental purification, but its efficient regulation remains difficult because oxygen activation, interfacial electron transfer, and molecular bond activation must be matched at the same catalytic interface [1]. Transition‐metal oxides are attractive for such processes because their variable metal valence states and lattice oxygen dynamics can participate directly in interfacial redox cycles [2, 3]. Single‐component oxides usually cannot balance activity, selectivity, and stability at the same time [4]. Redox‐active oxides can supply labile oxygen species, but their high reactivity may compromise selectivity and structural robustness. More stable oxygen‐buffering oxides provide better oxygen storage and release, but often show sluggish low‐temperature activation [5, 6]. This trade‐off makes mixed oxides attractive for integrating redox activity with oxygen‐buffering capacity. Cerium‐manganese oxides are a typical example of such mixed redox systems [7, 8]. Mn species contribute flexible valence cycling and low‐temperature redox activity, whereas CeO_2_ provides a stable fluorite framework and oxygen storage‐release capacity [9, 10, 11, 12]. In most CeMnO*_x_* studies, catalyst optimization has centered on the overall Mn/Ce ratio, phase composition, and long‐range structural order [13, 14]. These factors are important, but they cannot fully describe the local structure of the catalytically exposed surface. The role of short‐range surface composition, especially the near‐surface arrangement of MnO*_x_* and CeO_2_, has therefore remained largely unresolved.

This distinction is important because the reaction occurs at the exposed surface and near‐surface region, not in the bulk‐averaged composition of the oxide. The local arrangement of MnO*_x_* and CeO_2_ at these regions controls how oxygen is activated and how reactants interact with the catalyst. Conventional aqueous or solvent‐phase methods, including coprecipitation, citric acid complexation, and solution combustion, can adjust the phase composition, dispersion, and crystallinity of Mn–Ce oxides. However, they offer limited control over the vertical distribution of Mn and Ce species from the surface to the subsurface [8, 15]. Previous studies of Mn surface segregation in ceria‐based oxides and cation exsolution from perovskites have shown that cation distributions in oxides can be altered by composition and thermal or redox history [16, 17, 18, 19, 20]. However, spontaneous Mn segregation and conventional perovskite exsolution differ from the present objective of postsynthetically modifying the near‐surface distribution of oxidic Mn species in a preformed Mn–Ce oxide [21, 22]. As a result, Mn species are often incorporated into, highly dispersed within, or strongly bound to a CeO_2_‐dominated matrix, making it difficult to deliberately enrich MnO*_x_* at the outer surface. Although the ceria framework and Mn–Ce interaction are useful for bulk redox properties and structural stability, they may also reduce the accessibility of surface Mn‐derived redox sites. These considerations suggest a potential advantage of an Mn‐enriched surface structure, in which a MnO*_x_*‐enriched outer surface exposes Mn‐derived redox sites while the preserved CeO_2_ framework underneath retains its oxygen‐buffering function. These considerations suggest a potential advantage of a Mn‐enriched near‐surface configuration, in which the near‐surface Mn abundance is increased while the fluorite CeO_2_ framework is largely retained, thereby modifying the local Mn–Ce–O redox environment.

Here, we use sequential vacuum annealing followed by reduced‐pressure O_2_ treatment (the sequential vacuum‐O_2_ treatment) to convert the as‐prepared parent catalyst (N‐MnCe) into the corresponding surface‐reconstructed catalyst (R‐MnCe), featuring an Mn‐enriched near‐surface configuration while largely preserving the CeO_2_ framework. The sequential vacuum‐O_2_ treatment increases the near‐surface abundance of Mn‐containing species and produces an Mn‐enriched surface environment. By adjusting the treatment temperature, pressure, and oxidation time, we obtained R‐MnCe with a surface Mn/Ce ratio of 1.25 ± 0.04. Compared with N‐MnCe, R‐MnCe achieved 95.5% NO*_x_* conversion at 100°C and complete chlorobenzene (CB) conversion at 200°C under coupled removal conditions. Spectroscopic results and calculations suggest that the Mn‐enriched near‐surface environment modifies interfacial electron distribution, facilitates oxygen activation and the turnover of activated oxygen intermediates, and strengthens oxygen‐mediated kinetic interactions between NH_3_‐SCR and CB oxidation. These results indicate that the catalytic behavior of Mn–Ce oxides is not determined solely by the overall Mn/Ce composition, but can also be strongly influenced by the near‐surface distribution and local coordination of Mn and Ce species.

## 2 Results and Discussion

### 2.1 Vacuum‐Induced Surface Reconstruction and MnO_*x*_ Enrichment in Mn–Ce Oxides

R‐MnCe was constructed from N‐MnCe through the sequential vacuum‐O_2_ treatment (Figure 1a and Supporting Information). Under vacuum, lattice oxygen release creates a strong oxygen chemical potential gradient and induces the partial reduction of Mn species embedded in the CeO_2_‐dominated matrix. Compared with the robust Ce–O–Ce framework in fluorite CeO_2_, Mn–O–Mn and Mn–O–Ce linkages are more redox‐labile and more susceptible to oxygen removal under reducing conditions [23]. The weakening of these Mn‐associated bonds may generate local lattice distortion and facilitate the outward redistribution of Mn species from the subsurface or bulk region toward the surface. Subsequent O_2_ exposure re‐oxidizes and stabilizes the migrated Mn species as surface‐enriched MnO*_x_* configurations. This reconstructed architecture is described as an Mn‐enriched near‐surface configuration, characterized by increased Mn concentration in the outer region while the underlying CeO_2_ framework remains preserved.

**FIGURE 1:**
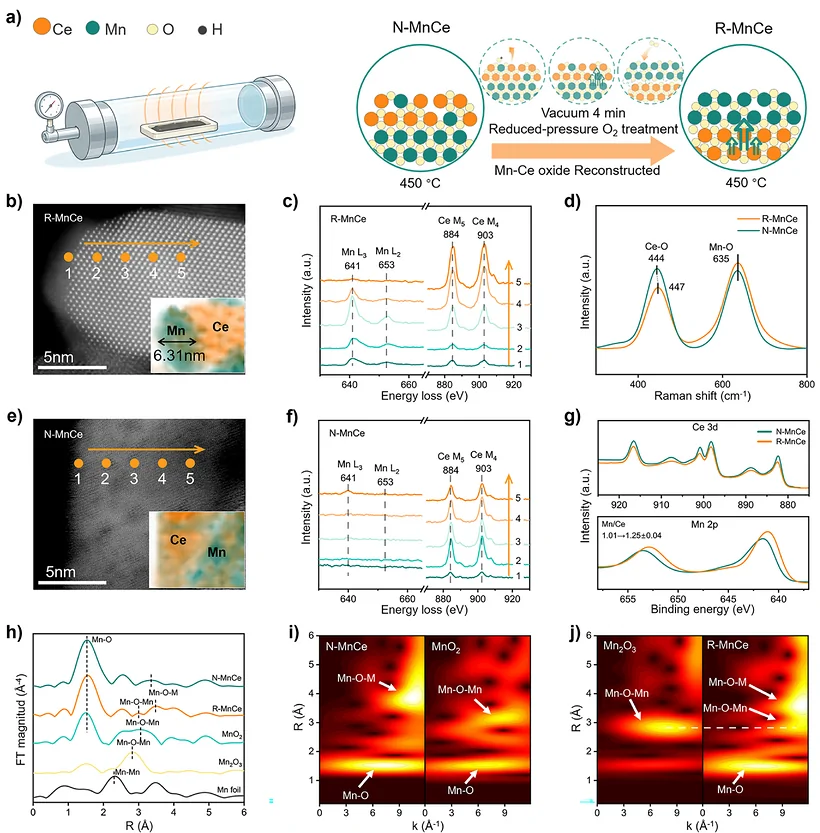
Vacuum‐Induced Surface Reconstruction and MnO*_x_* Enrichment. (a) The preparation process of reconstructed Mn‐Ce oxide. (b) HAADF‐STEM image of R‐MnCe sample and the corresponding EELS mapping. (c) Mn L_2,3_ edge and Ce M_4,5_ edge EELS spectra of R‐MnCe samples. (d) Raman spectrum of N‐MnCe and R‐MnCe catalyst. (e) HAADF‐STEM image of N‐MnCe sample and the corresponding EELS mapping. (f) Mn L_2,3_ edge and Ce M_4,5_ edge EELS images of N‐MnCe samples. (g) XPS spectra of N‐MnCe and R‐MnCe catalyst. (h) Fourier‐transform (FT) Mn K‐edge EXAFS spectra of N‐MnCe, R‐MnCe and reference compounds. (i)Wavelet transforms for the *k* ^3^‐weighted Mn K‐edge EXAFS signals of N‐MnCe and MnO_2_. (j) Wavelet transforms for the *k* ^3^‐weighted Mn K‐edge EXAFS signals of R‐MnCe and Mn_2_O_3_.

To probe this near‐surface reconstruction, we examined the phase evolution and Mn distribution before and after the sequential vacuum‐O_2_ treatment. XRD patterns showed that both N‐MnCe and R‐MnCe mainly retained the fluorite CeO_2_ structure (PDF#01‐073‐6318), indicating that the ceria framework was largely preserved after reconstruction (Figure S1). For R‐MnCe, additional weak reflections at approximately 33° and 40° could be assigned to Mn_2_O_3_, implying the formation of MnO*_x_*‐related domains after Mn redistribution (Figure S2). Whole‐pattern fitting showed that the relative XRD‐detectable Mn_2_O_3_ contribution increased from 18.2% in N‐MnCe to 45.0% in R‐MnCe (Figure S3). Because the fitted fractions do not account for amorphous or poorly crystalline MnO*_x_*, they are used as semiquantitative supporting evidence rather than as absolute phase contents. HAADF‐STEM further provided direct structural evidence. In N‐MnCe, lattice fringes corresponding to MnO_2_ (101) and CeO_2_ (111) were mainly observed within the bulk region (Figure S4), whereas R‐MnCe exhibited Mn_2_O_3_ (222) lattice fringes at the particle edge while retaining CeO_2_ lattice planes (Figure S5). These observations suggest that the treatment does not destroy the CeO_2_ framework, but drives the preferential enrichment of MnO*_x_* species at the outer surface.

The surface enrichment of Mn species was further confirmed by EELS analysis collected from the outermost surface toward the subsurface region (Figure 1b–f). In R‐MnCe, clear Mn L_2,3_‐edge signals were detected in the near‐surface regions, a locally observed Mn‐enriched region with an estimated thickness of approximately 6.31 nm. In contrast, the Mn signal in N‐MnCe was much weaker in the outer region and remained extremely weak with only a slight increase toward the innermost region, consistent with a Ce‐dominated surface and bulk‐confined Mn distribution. Further EELS mapping visually confirmed this reconstruction, revealing that Mn transitioned from a dispersed bulk state to an enriched outer surface (insets of Figure 1b,e). Fourier‐transform Mn K‐edge EXAFS and wavelet transform analyses showed a distinct scattering feature at approximately 2.7 Å in R‐MnCe, which is consistent with Mn–O–Mn coordination in Mn_2_O_3_‐like structures (Figure 1h–j). These results indicate that surface reconstruction generates surface‐enriched MnO*_x_* domains rather than isolated Mn species, establishing a reconstructed near‐surface architecture.

Complementary Raman and XPS analyses further confirmed the surface reconstruction. Raman spectra showed a weakened Ce–O F_2g_ vibration at 444 cm^−1^ and an enhanced Mn–O vibration at 635 cm^−1^ for R‐MnCe, indicating an increased contribution from surface Mn–O structures (Figure 1d) [24, 25]. XPS analysis showed that the Ce 3d signal decreased while the Mn 2p signal increased after treatment, with the calculated surface Mn/Ce atomic ratio exhibiting a corresponding increase from 1.01 in N‐MnCe to 1.25 in R‐MnCe. (Figure 1g). Meanwhile, the Mn 2p peak shifted toward lower binding energy, suggesting a shift of Mn species toward lower valence states during reconstruction. In situ DRIFTS during the treatment process further captured dynamic surface evolution, where hydroxyl signals changed under vacuum and oxygen re‐exposure generated water‐related bands and activated interfacial oxygen species (Figure S8). Collectively, these results demonstrate that sequential vacuum‐O_2_ treatment converts a Ce‐dominated Mn‐Ce oxide surface into a MnO*_x_*‐enriched reconstructed surface supported by a preserved CeO_2_ framework. Having established this surface MnO*_x_* enrichment, we next examined whether the surface compositional reconstruction also altered the electronic properties of the reconstructed interface [26].

### 2.2 Surface‐Enriched MnO_*x*_ Interfaces Enable Interfacial Electron Enrichment

The sequential vacuum‐O_2_ treatment reconstructed the initially Ce‐dominated surface into a MnO*_x_*‐rich surface, which was expected to substantially alter the interfacial electronic structure. To clarify this electronic reconstruction, DFT calculations were performed on N‐MnCe and R‐MnCe models representing idealized local Ce‐rich and Mn‐rich surface configurations (Figure S12). Density of states analysis showed that R‐MnCe exhibited stronger overlap between d and p states near the Fermi level, indicating enhanced p‐d orbital hybridization at the reconstructed surface MnO*_x_*/CeO_2_ interface (Figure 2a) [27]. Such stronger orbital coupling indicates that the MnO*_x_* species enriched on R‐MnCe expose interfacial sites that are more favorable for charge redistribution.

**FIGURE 2:**
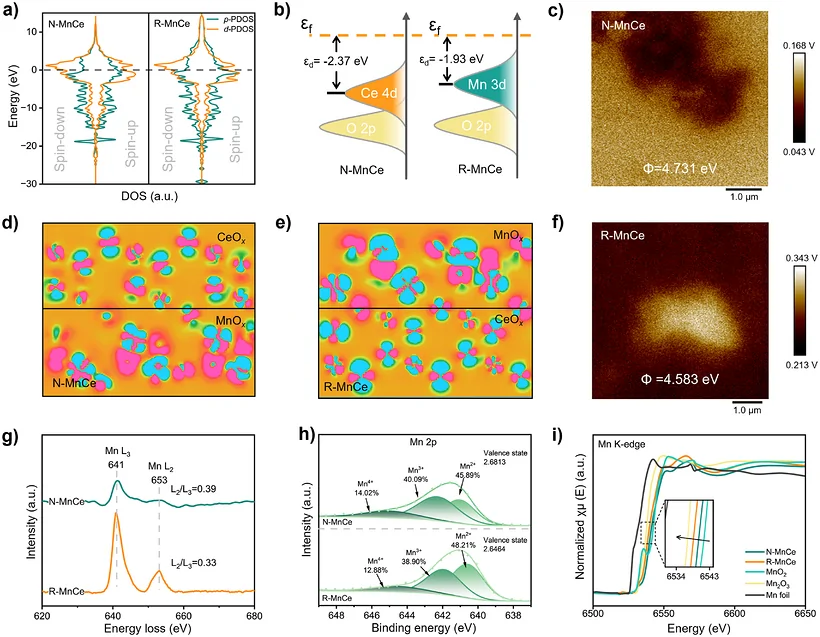
Electron‐rich surface MnO*_x_* interfaces induced by surface reconstruction. (a) Density of states (DOS) of N‐MnCe and R‐MnCe. (b) The crystal field splitting of *d* orbitals in electron configuration of Ce/Mn in different orbitals for N‐MnCe and R‐MnCe. (c) KPFM surface‐potential map of N‐MnCe calibrated using HOPG as the reference. (d) Electron localization function (ELF) of N‐MnCe (e) Electron localization function (ELF) of R‐MnCe. (f) KPFM surface‐potential map of R‐MnCe calibrated using HOPG as the reference. (g) Mn L_2,3_‐edge EELS spectra and L_2_/L_3_ white‐line intensity ratios of N‐MnCe and R‐MnCe. (h) XPS Mn 2p spectra of N‐MnCe and R‐MnCe catalyst. (i) Normalized Mn K‐edge XANES spectra of N‐MnCe, R‐MnCe, and reference compounds. The color scales were independently adjusted for each map to visualize local surface‐potential variations; therefore, the map colors should not be directly compared between the two samples.

The enhanced electronic activity was further supported by the upward shift of the d‐band center from −2.37 eV for N‐MnCe to −1.93 eV for R‐MnCe, approaching the Fermi level (Figure 2b) [26, 28]. This shift indicates a stronger interaction between surface Mn sites and adsorbed species, which is beneficial for electron transfer during oxygen activation and reactant transformation. In addition, Kelvin probe force microscopy (KPFM) measurements, which showed that the surface potential decreased from 4.731 eV for N‐MnCe to 4.583 eV for R‐MnCe (Figure 2c,f), suggesting that electrons on the reconstructed surface can be more readily transferred to adsorbed oxygen or reactant molecules [15]. Consistently, the electron localization function maps showed stronger electron accumulation around the surface‐enriched MnO*_x_* species in R‐MnCe, further supporting the formation of an electron‐rich MnO*_x_* interface after surface reconstruction (Figure 2d,e).

The DFT‐derived electron enrichment was further supported by the experimentally observed shift of Mn species toward lower valence states. Mn K‐edge XANES showed that the absorption edge of R‐MnCe shifted toward the lower‐energy region and was located between the Mn_2_O_3_ and MnO_2_ references, indicating a decrease in the average Mn oxidation state after reconstruction (Figure 2i). Mn L_2,3_‐edge EELS further showed that the L_2_/L_3_ white‐line intensity ratio decreased from 0.39 for N‐MnCe to 0.33 for R‐MnCe, suggesting that Mn species on the reconstructed surface shifted toward a lower‐valence state (Figure 2g). Consistently, Mn 2p XPS fitting revealed that the average Mn oxidation state decreased by 0.04 for R‐MnCe (Figure 2h) [29]. A weak six‐line hyperfine feature consistent with Mn(II) was observed for both R‐MnCe and N‐MnCe (Figure S17) [30].

Taken together, the theoretical and experimental results demonstrate that surface reconstruction does not merely induce surface Mn enrichment, but also reconstructs the electronic structure of the surface MnO*_x_*/CeO_2_ interface. Because oxygen activation in transition metal oxides is strongly governed by interfacial electron transfer, the enhanced electron‐donating capability of R‐MnCe is expected to directly influence oxygen mobility, activation, and participation under reaction conditions.

### 2.3 Electron‐Rich Interfaces Promote Oxygen Activation and Participation

Based on the surface MnO*_x_* enrichment and interfacial electron reconstruction demonstrated above, we next investigated whether the electron‐rich surface MnO*_x_* interface could facilitate oxygen release, activation, and participation [31]. O_2_‐TPD and H_2_‐TPR were first performed to evaluate the releasable oxygen species and reducibility of N‐MnCe and R‐MnCe [32]. The O_2_‐TPD profiles showed multiple oxygen desorption features for both samples (Figure 3a). The peaks at 150°C–350°C can be assigned to weakly adsorbed oxygen and chemisorbed oxygen species, whereas the higher‐temperature signals are associated with lattice oxygen species [33, 34]. Compared with N‐MnCe, R‐MnCe exhibited a more pronounced high‐temperature desorption signal, suggesting an increased amount of releasable lattice‐oxygen‐related species that may participate in interfacial redox cycling. Consistently, the H_2_‐TPR profile of R‐MnCe shifted distinctly toward lower temperature (Figure 3b). The reduction feature around 193°C demonstrates that these oxygen species are more readily activated and consumed, highlighting the enhanced low‐temperature redox capability [35].

**FIGURE 3:**
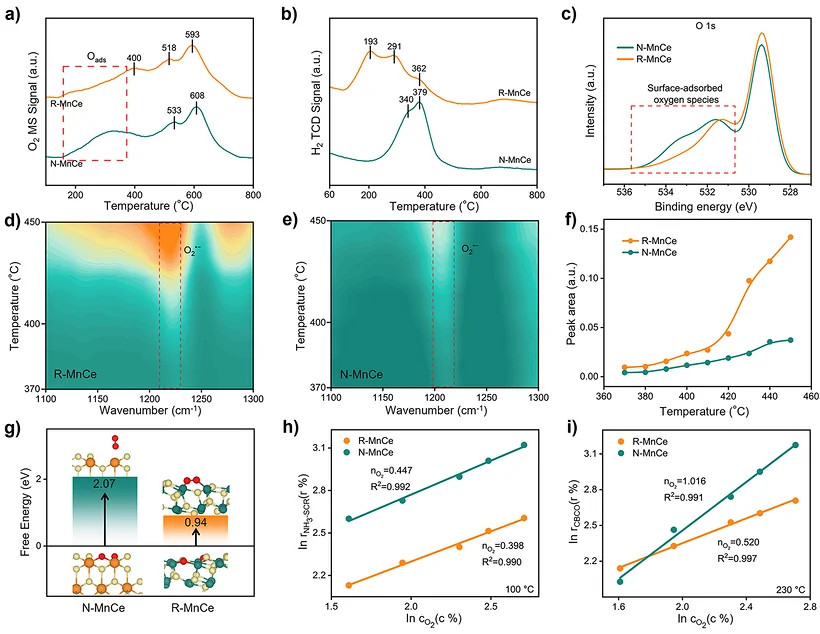
Surface MnOx enrichment promotes oxygen activation and participation. (a) O_2_−TPD profiles of N‐MnCe and R‐MnCe. (b) H_2_‐TPR profiles of N‐MnCe and R‐MnCe. (c) XPS O1s spectra of N‐MnCe and R‐MnCe catalyst. In situ DRIFTS spectra of superoxide species over (d) R‐MnCe and (e) N‐MnCe during temperature‐programmed desorption under Ar after O_2_ adsorption at 370°C. (f) Integrated peak area of the superoxide‐related activated oxygen band at 1220 cm^−1^ as a function of temperature for N‐MnCe and R‐MnCe. (g) Free‐energy comparison for the formation of superoxide‐related activated oxygen intermediates over N‐MnCe and R‐MnCe. (h) Apparent reaction orders with respect to O_2_ for NH_3_‐SCR during the coupled elimination of NO*_x_* and CB over N‐MnCe and R‐MnCe at 100°C. (i) Apparent reaction orders with respect to O_2_ for CBCO during the coupled elimination of NO*_x_* and CB over N‐MnCe and R‐MnCe at 230°C.

The oxygen chemical environment was further examined by O1s XPS. R‐MnCe showed an increased contribution from lattice‐oxygen‐related components in O1s XPS, suggesting a modified oxygen chemical environment after surface reconstruction (Figure 3c). This behavior is consistent with the formation of surface‐enriched, electron‐rich MnO*_x_* species, which can facilitate electron transfer to oxygen species and promote their activation. Together with the above electronic‐structure results, this modified oxygen environment suggests that oxygen activation over R‐MnCe is promoted by both surface MnO*_x_* enrichment and electronic reconstruction at the surface MnO*_x_*/CeO_2_ interface. Ex situ O 1s XPS and EPR analyses of R‐MnCe after parallel reactions conducted with and without gaseous O_2_ showed greater lattice‐oxygen depletion and a stronger defect‐related signal under O_2_‐free conditions, providing complementary but indirect evidence for lattice‐oxygen consumption and O_2_‐assisted replenishment (Figures S16 and S18). Consistently, the calculated barrier for O_2_‐assisted vacancy replenishment was substantially lower on R‐MnCe than on N‐MnCe (0.47 vs. 1.48 eV), supporting a more readily sustained oxygen cycle at the reconstructed MnO*_x_*‐enriched interface.

To directly monitor the evolution of activated oxygen intermediates, in situ DRIFTS was conducted during temperature‐programmed treatment after O_2_ adsorption (Figure 3d,e) [23, 26]. A distinct band at 1220 cm^−1^, assigned to superoxide‐related activated oxygen intermediates, appeared on R‐MnCe during heating, whereas only a weak response was observed on N‐MnCe [36]. The integrated area of this band increased continuously on R‐MnCe with increasing temperature (Figure 3f), indicating that the reconstructed MnO*_x_*‐rich interface favors the formation and thermal persistence of activated oxygen species. Consistently, free‐energy calculations showed that the energy required to generate activated oxygen intermediates decreased from 2.07 eV on N‐MnCe to 0.94 eV on R‐MnCe (Figure 3g). These results suggest that surface reconstruction lowers the oxygen activation barrier by constructing an electron‐rich MnO*_x_* interface.

The participation of oxygen species under reaction conditions was further evaluated by determining the apparent reaction orders with respect to O_2_ for NH_3_‐SCR and CBCO during the coupled reaction (Figure 3 h,i) [37]. The O_2_ reaction order for NH_3_‐SCR changed only slightly from 0.45 on N‐MnCe to 0.40 on R‐MnCe, whereas that for CBCO decreased substantially from 1.01 to 0.52. The lower apparent O_2_ reaction order indicates that the reaction becomes less dependent on gaseous O_2_, suggesting a larger contribution from preactivated interfacial oxygen or lattice oxygen mediated redox processes. This change is consistent with a greater contribution from a Mars‐van Krevelen‐type pathway involving surface or lattice oxygen, although the apparent reaction order alone does not establish a complete mechanistic transition. Together, these TPD, TPR, XPS, in situ DRIFTS, kinetic, and DFT results demonstrate that the reconstructed electron‐rich MnO*_x_* interface facilitates oxygen release, activation, regeneration, and participation, laying the foundation for enhanced molecular activation.

### 2.4 Catalytic and Kinetic Evidence for Oxygen‐Mediated NO_*x*_/CB Coupling

After confirming the enhanced oxygen release, activation, and participation over R‐MnCe, we further examined whether these oxygen‐related properties could translate into improved NH_3_ activation, CB oxidation, and coupled redox performance. Based on the optimized treatment conditions (Figures S27–S34), the R‐MnCe sample obtained by the sequential vacuum‐O_2_ treatment at 450°C for 4 min showed the most pronounced activity enhancement. In the individual NH_3_‐SCR reaction, both N‐MnCe and R‐MnCe exhibited volcano‐shaped NO*_x_* conversion profiles, with the maximum conversion appearing at around 200°C (Figure 4a). At this temperature, the NO*_x_* conversion increased from 87.9% over N‐MnCe to 99.8% over R‐MnCe, demonstrating that surface reconstruction significantly improves low‐temperature NH_3_‐SCR activity. The individual CBCO reaction was also promoted after structural reconstruction. At 300°C, CB conversion increased from 71.1% over N‐MnCe to complete conversion over R‐MnCe (Figure 4b). Moreover, CB‐TPSR showed that CO_2_ evolution over R‐MnCe shifted toward lower temperature, with a distinct low‐temperature CO_2_ signal appearing at 179°C (Figure 4c) [38]. These results indicate that the surface‐enriched MnO*_x_* species facilitate CB activation and promote its deep oxidation to CO_2_.

**FIGURE 4:**
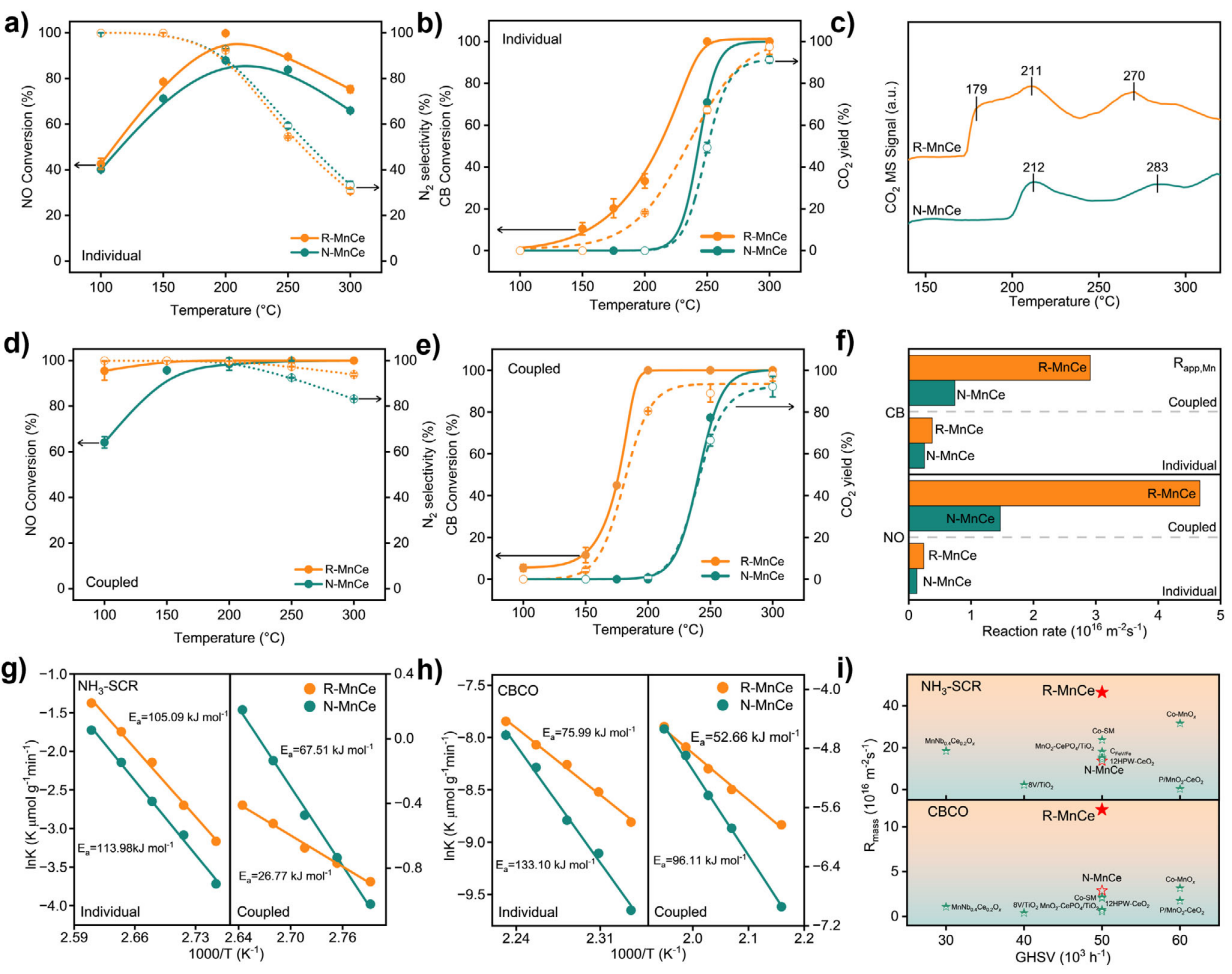
Catalytic performance in individual and coupled NO*_x_*‐CB Removal. (a) NO conversion and N_2_ selectivity during individual NH_3_‐SCR. (b) CB conversion and CO_2_ yield during individual CBCO. (c) CB‐TPSR profiles of N‐MnCe and R‐MnCe. (d) NO conversion and N_2_ selectivity during coupled NO*_x_*‐CB removal. (e) CB conversion and CO_2_ yield during coupled NO*_x_*‐CB removal. (f) Mn‐content‐corrected BET‐area‐normalized apparent reaction rates R_app, Mn_ of N‐MnCe and R‐MnCe for NH_3_‐SCR at 150°C and CBCO at 200°C under individual and coupled conditions. (g) *E*_a_ of N‐MnCe and R‐MnCe catalyst in NH_3_‐SCR. (h) *E*_a_ of N‐MnCe and R‐MnCe catalyst in CBCO. (i) Comparison of R_mass, NO_ with other catalysts at 150°C and R_mass, CB_ with other catalysts at 200°C. Apparent mass‐normalized rates as a function of GHSV. Error bars in figure a–b and d–e represent the standard deviation from three independent measurements (n = 3).

Having established the enhanced activity in the individual reactions, we next evaluated whether the surface‐enriched MnO*_x_* interface could further couple NO*_x_* reduction and CB oxidation under cofeeding conditions through oxygen‐mediated interfacial chemistry [34]. Under coupled NO*_x_*/CB removal conditions, R‐MnCe showed much higher NO*_x_* conversion than N‐MnCe. Under anhydrous conditions, R‐MnCe achieved 95.5% NO*_x_* conversion at 100°C, whereas N‐MnCe reached only 64.1%. Notably, the presence of CB markedly improved the N_2_ selectivity of R‐MnCe at medium and high temperatures. Even at 300°C, R‐MnCe maintained 93% N_2_ selectivity under coupled reaction conditions, in sharp contrast to only about 30% in the individual NH_3_‐SCR reaction. This behavior provides an important indication that CB oxidation can influence the NH_3_‐SCR pathway through shared interfacial oxygen chemistry. After introducing 5 vol.% H_2_O, R‐MnCe still maintained a broad efficient NO*_x_* removal window of 162°C–255°C with N_2_ selectivity above 90% (Figures 4d, S19, and S21). Meanwhile, CB oxidation was also strongly promoted. At 200°C, N‐MnCe showed almost no CB conversion, while R‐MnCe achieved complete CB conversion with a higher CO_2_ yield, indicating that the surface‐enriched MnO*_x_* interface simultaneously accelerates CB activation and deep oxidation under coupled conditions (Figures 4e, S32, and S34). Stepwise and reference‐sample controls showed that the characteristic multistep reduction behavior and enhanced coupled activity of R‐MnCe arose from the sequential vacuum‐O_2_ treatment sequence rather than any individual treatment step, conventional impregnation, or physical mixing (Figures S24–S27 and S39–S40).

To account for both surface‐area and surface‐Mn‐content effects, normalized apparent reaction rates and apparent activation energies were compared. Although the specific surface area of N‐MnCe and R‐MnCe remained essentially unchanged after surface reconstruction (Table S1), R‐MnCe exhibited higher reaction rates than N‐MnCe. The BET‐area‐normalized rate was further corrected using the XPS‐derived Mn/(Mn + Ce + O) fraction, yielding R_app, Mn_ (Figure 4f). Under individual reaction conditions, the R_app, Mn_ ratios of R‐MnCe to N‐MnCe were 1.86 for NO and 1.49 for CB; under coupled conditions, the corresponding ratios were 3.18 and 3.93, respectively [37, 39]. Kinetic analysis further showed that the apparent activation energy for CBCO decreased from 133.10 kJ/mol over N‐MnCe to 75.99 kJ/mol over R‐MnCe, and further to 52.66 kJ/mol under coupled conditions. For NH_3_‐SCR, the apparent activation energy decreased from 113.98 to 105.09 kJ/mol after surface reconstruction and further dropped to 26.77 kJ/mol during the coupled reaction [37]. The similar N_2_ selectivities and apparent activation energies for individual NH_3_‐SCR, together with the higher Mn‐content‐corrected R_app, Mn_ of R‐MnCe, indicate that the dominant pathway is retained and that the enhanced activity reflects contributions from accessible Mn‐containing species and modified interfacial redox properties rather than a new active‐site type. Although coupled feeding promoted the catalytic performance of both N‐MnCe and R‐MnCe, the enhancement was substantially more pronounced over R‐MnCe. This difference suggests that NO*_x_* reduction and CB oxidation are not simply two independent parallel reactions and that the MnO*_x_*‐enriched surface of R‐MnCe further facilitates their kinetic coupling through oxygen‐mediated interfacial processes. Overall, the performance and kinetic results indicate that the reconstructed MnO*_x_*‐enriched surface increases the accessibility of redox‐active Mn species and promotes oxygen‐mediated molecular activation. Rather than creating the coupling effect exclusively, this surface reconstruction strengthens the interaction between the NH_3_‐SCR and CB oxidation pathways, as further examined by in situ DRIFTS and MES‐DRIFTS.

### 2.5 Spectroscopic Evidence for Oxygen‐mediated Coupling Between NH_3_ Activation and CB Oxidation

To clarify how the MnO*_x_*‐enriched reconstructed surface couples NH_3_‐SCR with CB oxidation, in situ DRIFTS was first used to monitor the evolution of surface species during sequential exposure to NH_3_, NO, and CB at 200°C (Figure 5a,b). Compared with N‐MnCe, R‐MnCe showed weaker L‐NH_3_ (1250 cm^−1^) and B‐NH_3_ (3258, 3373 cm^−1^) signals but stronger bands associated with activated ─NH_2_ species and H_2_O formation, indicating that the MnO*_x_*‐enriched interface favors NH_3_ activation and dehydrogenation rather than simple NH_3_ accumulation [16, 40, 41, 42, 43, 44]. After CB introduction, the L‐NH_3_ signal was rapidly consumed, accompanied by the appearance of CO_2_‐related bands, indicating that CB oxidation occurred concurrently with the consumption of activated NH_3_ species. This synchronous evolution suggests that NH_3_ activation and CB oxidation are not independent surface events on R‐MnCe, but are kinetically linked through oxygen‐mediated interfacial redox chemistry.

**FIGURE 5:**
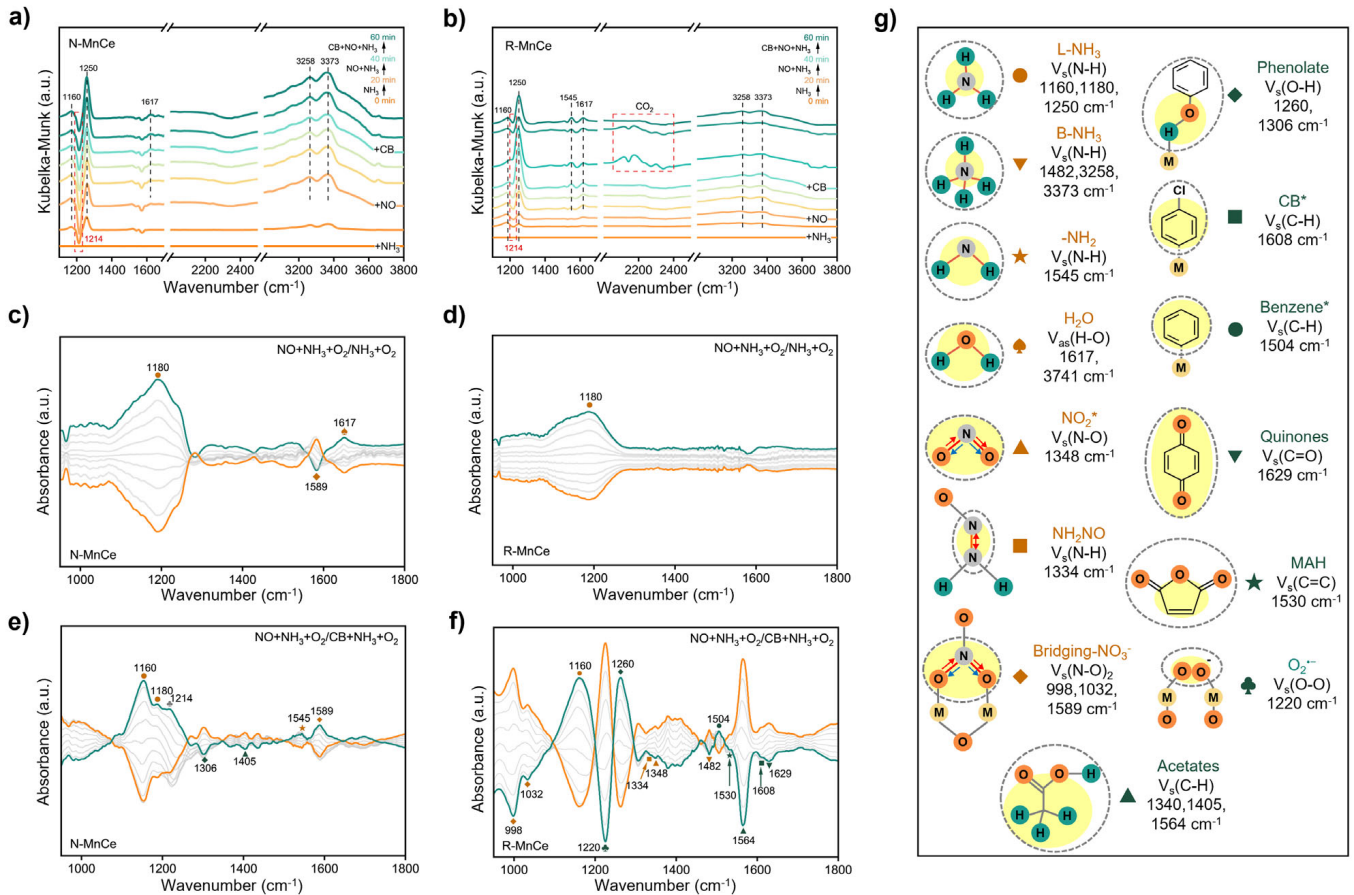
Spectroscopic evidence for oxygen‐mediated coupling between NH_3_ activation and CB oxidation. In situ DRIFTS spectra of surface species evolution over (a) N‐MnCe and (b) R‐MnCe during sequential exposure to NH_3_, NO, and CB at 200°C. MES‐DRIFTS spectra of (c) N‐MnCe and (d) R‐MnCe during NO+NH_3_+O_2_/NO+O_2_ modulation experiment at 200°C. MES‐DRIFTS spectra of (e) N‐MnCe and (f) R‐MnCe during NO+NH_3_+O_2_/CB+NH_3_+O_2_ modulation experiment at 200 °C. (g) Schematic diagram of surface species and their corresponding infrared vibration bands (yellow shaded areas indicate vibration positions).

Gas‐switching experiments further clarified the role of the MnO*_x_*‐enriched reconstructed surface in regulating CB‐derived intermediates (Figures S10 and S11) [43, 45, 46, 47]. On N‐MnCe, phenolate (1260 cm^−1^) and acetates (1340 cm^−1^) species accumulated evidently, indicating that aromatic or oxygenated intermediates derived from CB were more likely to remain on the surface. In contrast, R‐MnCe showed a much weaker phenolate signal, whereas acetate‐related species were more transient and decreased rapidly after introducing NH_3_ and NO. This behavior suggests that the MnO*_x_*‐enriched reconstructed surface promotes the cleavage of phenolate intermediates into smaller oxygenated species, thereby facilitating deep oxidation and minimizing the accumulation of CB‐derived residues. However, the bands associated with Mn‐OH or activated oxygen intermediates overlap with those of L─NH_3_, phenolate, ─NH_2_, and acetate species. Therefore, conventional DRIFTS alone cannot fully resolve the dynamic evolution of these coupled intermediates.

To overcome this limitation, modulation excitation phase‐sensitive detection DRIFTS (MES‐DRIFTS) was further employed to distinguish kinetically responsive surface species during periodic gas modulation (Figure 5c,d) [40, 48, 49]. This method separates overlapping signals in the phase domain according to differences in response kinetics and effectively filters out static background contributions. In the control experiment using NO pulses under an O_2_ and NH_3_ atmosphere, only L─NH_3_ and nitrate‐related species were clearly detected on both N‐MnCe and R‐MnCe, whereas no obvious ─NH_2_ species or activated oxygen intermediates appeared. The absence of dynamically responsive ─NH_2_ and activated oxygen intermediates indicates that NO modulation alone does not generate the same coupled surface response under the present conditions.

Alternating the introduction of NO and CB under NH_3_/O_2_ elicited markedly different surface responses on N‐MnCe and R‐MnCe. On N‐MnCe, the accumulation of inert nitrates, phenolates, and acetates suggests a sluggish conversion of both nitrogenous and aromatic intermediates. Notably, the signal at 1214 cm^−1^ should be assigned to the positive response of Mn─OH species rather than the negative consumption of activated oxygen intermediates [45]. In contrast, R‐MnCe generated distinct dynamic signals assignable to superoxide‐related activated oxygen intermediates (1220 cm^−1^), NH_2_NO (1334 cm^−1^), adsorbed NO_2_ (1348 cm^−1^), maleate (1530 cm^−1^), adsorbed CB (1504 cm^−1^), and benzoquinone (1629 cm^−1^) [43, 46, 50]. These results suggest that, under the present modulation conditions, CB modulation redirects the surface oxygen‐mediated pathway on the reconstructed MnO*_x_*‐enriched interface, activating oxygen‐mediated CB oxidation while simultaneously promoting NH_3_ activation and NH_2_NO formation. Therefore, the enhanced coupled removal of NO*_x_* and CB over R‐MnCe can be attributed to an oxygen‐mediated interfacial coupling process that links NH_3_ activation, CB dechlorination, and deep oxidation pathways.

### 2.6 Interfacial Electron Transfer Enables Oxygen‐Mediated Reaction Coupling

The spectroscopic results above suggest that the coupled reaction over R‐MnCe involves dynamically formed activated oxygen intermediates, NH_2_NO species, and CB‐derived oxygenates. To rationalize the energetic origin of this pathway coupling, DFT calculations were further performed to examine oxygen activation/regeneration, reactant adsorption, and CB electronic activation on the reconstructed MnO*_x_* interface. The free‐energy evolution of activated oxygen intermediate formation was calculated (Figure 6a) [23, 26]. On the surface‐enriched MnO*_x_* interface of R‐MnCe, the activation of lattice oxygen and its coupling with adjacent oxygen species require energy barriers of only 0.41 and 0.54 eV, respectively, indicating that the reconstructed interface can readily activate oxygen species. After the formation of activated oxygen intermediates, oxygen vacancy sites are generated on the reconstructed MnO*_x_* interface. The subsequent replenishment of these vacancies by gaseous O_2_ is the key step for sustaining the interfacial oxygen cycle. Notably, the energy barrier for O_2_‐assisted vacancy replenishment on R‐MnCe is only 0.47 eV, much lower than the 1.48 eV calculated for N‐MnCe. Overall, these calculations show that surface reconstruction substantially lowers the barriers for oxygen activation and regeneration, enabling a more efficient interfacial oxygen cycle for subsequent NH_3_‐SCR and CB oxidation.

**FIGURE 6:**
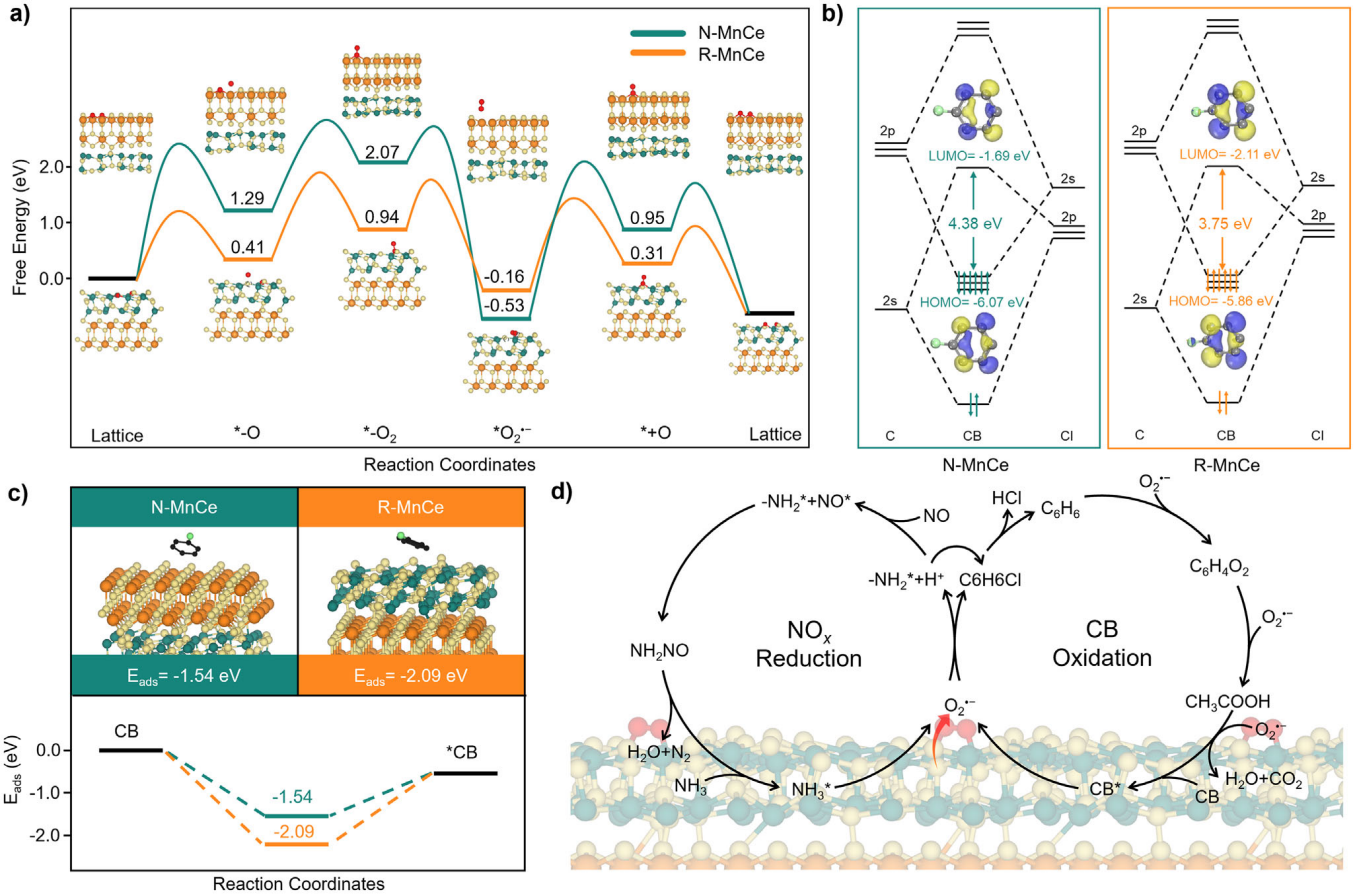
Interfacial electron transfer enables oxygen‐mediated reaction coupling. (a) The potential energy diagram for activated oxygen intermediate formation and regeneration for N‐MnCe and R‐MnCe. (b) Frontier orbital energy level distributions after CB adsorption on N‐MnCe and R‐MnCe catalysts. (c) Adsorption free energies of NO and CB on N‐MnCe and R‐MnCe. (d) Proposed oxygen‐mediated coupling mechanism for NH_3_‐SCR and CBCO by R‐MnCe. The atomic structures represent idealized local Ce‐rich and Mn‐rich surface configurations used to evaluate the effects of Mn surface enrichment.

The promoting effect of the reconstructed interface on reactant activation was further evaluated using CB as a representative molecule. Adsorption calculations indicate that R‐MnCe provides more favorable adsorption sites for CB, with the adsorption energy of CB from −1.54 to −2.09 eV (Figure 6c). In addition, frontier orbital analysis revealed that the HOMO‐LUMO gap of adsorbed CB decreased from 4.38 eV on N‐MnCe to 3.75 eV on R‐MnCe (Figure 6b). This narrowed energy gap indicates stronger electronic interaction between CB and the electron‐rich MnO*_x_* interface, implying a more activated electronic state of adsorbed CB that may facilitate subsequent C─Cl bond weakening. These results suggest that the surface reconstruction structure favors CB activation by enhancing adsorption and electronically polarizing the adsorbed CB molecule, which may facilitate subsequent C─Cl bond weakening, dechlorination, and ring‐opening oxidation.

Based on the experimental observations and DFT calculations, an oxygen‐mediated coupling mechanism is proposed for the coupled removal of NO*_x_* and CB over R‐MnCe (Figure 6d). The sequential vacuum‐O_2_ treatment‐induced MnO*_x_*‐enriched outer surface provides accessible redox‐active Mn sites and an electron‐rich interfacial environment that facilitates oxygen activation and regeneration of activated interfacial oxygen species. These activated oxygen intermediates participate in two interconnected pathways. In the NH_3_‐SCR pathway, the electron‐rich interface promotes NH_3_ activation to generate surface ─NH_2_ species, which further couples with adsorbed NO species to form the key NH_2_NO intermediate and finally decomposes into N_2_ and H_2_O. In the CB oxidation pathway, activated oxygen intermediates, assisted by surface hydrogen from NH_3_ activation, promote C─Cl bond weakening and subsequent HCl formation. Meanwhile, the CB‐derived aromatic intermediates undergo sequential oxidation into quinone, maleate, and acetate species prior to final mineralization to CO_2_ and H_2_O. Therefore, the enhanced coupled NO*_x_* and CB removal over R‐MnCe cannot be explained simply by the independent enhancement of NH_3_‐SCR and CBCO reactions, but arises from the oxygen‐mediated integration of multiple interfacial reaction steps. These results establish a structure‐electron‐oxygen‐reactivity relationship in which near‐surface reconstruction enriches electron‐rich MnO*_x_* interfaces on the preserved CeO_2_ framework, driving oxygen‐mediated molecular activation and reaction coupling.

## 3 Conclusions

In this work, we used a sequential vacuum‐O_2_ treatment to reconstruct Ce‐dominated Mn–Ce oxides into a surface reconstruction structure with MnO*_x_* enriched at the surface. During the treatment, part of the Mn species originally confined in the bulk migrated toward the surface, and the following oxygen exposure stabilized these Mn species on the preserved CeO_2_ framework. As a result, the surface changed from Ce‐dominated to MnO*_x_*‐enriched, providing more accessible Mn redox sites and a more electron‐rich interfacial environment. This surface reconstruction promoted oxygen activation and oxygen regeneration, which accounts for the improved activity of R‐MnCe in both NH_3_‐SCR and CB oxidation. Under coupled reaction conditions, R‐MnCe also maintained efficient NO*_x_* and CB removal in the presence of water. In situ spectroscopy and DFT calculations suggest that activated interfacial oxygen species are involved in NH_3_ activation, NO conversion, CB dechlorination, and further oxidation of CB‐derived intermediates. These linked steps accelerate intermediate turnover and promote CO_2_ formation. The results highlight near‐surface rearrangement of MnO*_x_* and CeO_2_ as a practical way to expose surface Mn redox sites and improve oxygen‐mediated coupled redox reactions.

## Author Contributions

Y.Y. and Y.J. proposed and designed the research plan and experimental scheme. Y.J., Y.Y. performed the characterization of the materials. Y.J. and Z.X. carried out the synthesis and the activity tests of the materials and data analysis. Y.J. and Y.Y. conducted mechanistic investigation experiments and analysis. Y.Y. performed the first‐principles calculations. Y.J., M.L., E.C., and Y.Y. cowrote the manuscript. M.L., E.C., and Y.S. supervised the work. All authors discussed the results and provided comments on the manuscript.

## Conflicts of Interest

The authors declare no conflicts of interest.

## Code Availability

All code used in the simulations supporting this paper is available from the respective authors upon request.

## Supporting information




**Supporting File**: anie74908‐sup‐0001‐SuppMat.doc.

## Data Availability

The data that support the findings of this study are available from the corresponding author upon reasonable request.
