# Supplementary material for: Vacuum‐Induced Surface Reconstruction of Mn–Ce Oxides for Low‐Temperature Coupled Redox Catalysis
Source: Angew Chem Int Ed Engl. 2026 Sep 21;65(45):e2739215. doi: 10.1002/anie.2739215 (PMC13725578; doi:10.1002/anie.2739215)
Supplement: Supplementary file 1 [file ANIE-65-e2739215-s001.doc]

# Supplementary Methods

# 1. Experimental and Characterization

## 1.1 Catalyst preparation

A solution of 5.74 g Mn(NO_3_)_2_·6H_2_O (99.9%, Macklin) and 8.68 g Ce(NO_3_)_3_·6H_2_O (99.9%, Aladdin) was prepared in 50 mL anhydrous ethanol (99.9%, Aladdin) under stirring until a homogeneous slurry was obtained (solution 1). Separately, 7.68 g citric acid (99.5%, Macklin) was dissolved in 40 mL anhydrous ethanol under stirring to form a homogeneous slurry (solution 2). Solution 1 and solution 2 were then mixed and stirred for 30 min, followed by drying at 60 °C for 5 h to induce gelation and subsequent drying at 100 °C for 12 h. The obtained solid was finally calcined in air at 400 °C for 2 h with a heating rate of 3 °C/min, yielding Normal MnCe (N-MnCe).

Then, 0.2 g of N-MnCe was placed in a tubular furnace. The reactor was evacuated until the pressure reached <1.3 kPa (variable 1), after which the sample was heated to 450 °C at a rate of 20 °C/min and held at this temperature for 25 min. Four minutes after reaching 450 °C (variable 2), pure O_2_ was introduced until the pressure increased to 51.3 kPa, thereby inducing the *in situ* exsolution of the Mn layer to the surface. The resulting sample was denoted as surface-reconstructed MnCe catalyst (R-MnCe).

Mo/Ce was prepared by an impregnation method. CeO_2_ was first obtained by calcining Ce(NO_3_)_3_·6H_2_O at 500 °C in air. Typically, ammonium molybdate (0.041 g, Macklin) was dissolved in deionized water and mixed with fluorite-structured CeO_2_ (0.97 g). The water was slowly removed using a rotary evaporator, and the obtained solid was dried at 100 °C overnight, followed by calcination at 500 °C for 3 h in air. The resulting catalyst was denoted as Mo/Ce. W/Ce was prepared using the same procedure, except that ammonium tungstate was used as the W precursor instead of ammonium molybdate.

For the treated samples, Mo/Ce and W/Ce were subjected to the same vacuum annealing followed by low-pressure oxygen treatment as that used for R-MnCe. The obtained samples were denoted as Mo/Ce-R and W/Ce-R, respectively.

CeO_2_ was prepared by calcining Ce(NO_3_)_3_·6H_2_O (99.9%, Aladdin) in air at 500 °C for 3 h. MnCe-Imp was prepared using the same conventional impregnation procedure as that used for Mo/Ce. Briefly, 3.33 g of Mn(NO_3_)_2_·6H_2_O (99.9%, Macklin) was dissolved in deionized water and mixed with 2.00 g of the as-prepared CeO_2_, corresponding to a nominal Mn/Ce molar ratio of 1:1. The water was slowly removed using a rotary evaporator, and the resulting solid was dried at 100 °C overnight and subsequently calcined in air at 500 °C for 3 h. The obtained catalyst was denoted as MnCe-Imp.

MnCe-PM was prepared by physically mixing Mn_2_O_3_ and CeO_2_ at a nominal Mn/Ce molar ratio of 1:1. Specifically, 1.069 g of Mn_2_O_3_ was thoroughly mixed with 2.331 g of the as-prepared CeO_2_. The resulting physical mixture was denoted as MnCe-PM.

## 1.2 High-Angle Annular Dark-Field (HAADF) Electron Microscope

HAADF images of the samples were obtained using a ThermoFisher Spectra 300 S/TEM transmission electron microscope with a convergence angle of 25 mrad and inner and outer collection angles of 59 and 200 mrad, respectively. To acquire the spectroscopic data needed for EELS elemental mapping, the aberration-corrected JEM ARM 200F scanning transmission electron microscopy system coupled with a cold field emission gun operating at 200 kV was utilized to examine the microstructure and determine the elemental distribution. The EDX characterization was performed via a JED2300 detector from JEOL. The collection semi-angle and probe convergence angle were found to be 54–220 and 20.6 mrad, respectively.

## 1.3 X-ray absorption fine structure (XAFS)

The XAFS measurements were conducted using the EasyXAFS300^+^ beamline, which is equipped with a high-resolution Roland ring-structured X-ray monochromator designed for X-ray absorption fine structure spectroscopy (XAFS). The monochromator operates within a Bragg angle range of 55° to 85°, corresponding to an energy range of 5–19 keV. At the terminal station, a one-element silicon drift detector with an effective area of 150 mm² is positioned downstream of the monochromator output and is utilized for both transmission and fluorescence-mode XAFS measurements. Data preprocessing, including background subtraction, pre-edge, and post-edge correction, was performed using Athena software (version 0.9.26). Fourier transform analysis was subsequently carried out using Artemis software (version 0.9.26). Wavelet transform analysis was conducted by importing χ(k) data exported from Athena into MATLAB code.

## 1.4 X-ray photoelectron spectroscopy (XPS)

The experiment was conducted using an X-ray photoelectron spectrometer (XPS). Monochromatic Al Kα radiation (hν = 1486.6 eV) was employed as the excitation source, with a beam spot diameter of 400 μm. The analyzer operated in constant analyzer energy (CAE) mode at a passing energy of 150.0 eV, and the energy step size was set to 1.000 eV during spectral acquisition, 50 ms dwell time, and charge correction with C 1s=284.80 eV binding energy standard.

## 1.5 Temperature-programmed reduction with H_2_ (H_2_-TPR)

The temperature-programmed reduction with H_2_ (H_2_-TPR) experiments were carried out on a chemisorption instrument. Before conducting the testing, the catalyst samples were subjected to a pre-treatment at 300°C for 60 minutes using helium as carrier gas. This pre-treatment was performed to remove moisture and impurities from the samples. (Micromeritics, AutoChem II 2920, USA).

## 1.6 Temperature-programmed desorption with O_2_ (O_2_-TPD)

Temperature-programmed desorption with O_2_ (O_2_-TPD)experiments were carried out on a Fixed-bed reactor-MS. Before conducting the testing, the catalyst samples were subjected to a pre-treatment at 300°C for 60 minutes using Argon as carrier gas. This pre-treatment was performed to remove moisture and impurities from the samples. (Hiden, DECRA, UK).

## 1.7 Temperature-programmed surface reaction with CB (CB-TPSR)

Temperature-programmed surface reaction with CB (CB-TPSR)experiments were carried out on a Fixed-bed reactor-MS with a reaction feed gas (100ppm CB, 5 vol % O_2_, Ar balance) and the samples were heated from 50 to 600 °C. Before conducting the testing, the catalyst samples were subjected to a pre-treatment at 300°C for 60 minutes using Argon as carrier gas. This pre-treatment was performed to remove moisture and impurities from the samples. (Hiden, DECRA, UK).

## 1.8 Characterization instruments

The nitrogen adsorption isotherm is performed on a physisorption instrument (Micromeritics, ASAP2020, USA). X-ray diffraction spectroscopy (XRD) is recorded using an X-ray diffractometer (Bruker, Bruker D8 Advance, Germany). Raman spectra were collected on a Raman spectrometer equipped with a 532 nm laser source and a Renishaw Centrus detector. The spectra were recorded in the range of 100.30–1998.50 cm⁻¹ with an estimated spectral resolution of ~1 cm⁻¹. Each spectrum was acquired with an exposure time of 60 s and 5 accumulations.

## 1.9 Measurement of NH_3_-SCR and CBCO activity and kinetics

The activity tests are performed with 200 mg catalyst within 40–60 meshes in a fixed-bed quartz reactor. The simulated flue gas consists of 100 ppm of CB, 500 ppm of NO, 500 ppm of NH_3_, 5 % of O_2_, balanced with Ar, with a gas hourly space velocity (GHSV) of 50000 h^-1^. The concentrations of NO_2_ and N_2_O were monitored by a Fourier-transformed infrared gas detector (ABB MBGAS-3000), NO and NH_3_ were monitored by MS (Hiden, DECRA), CB and CO_2_ were monitored by GC(FULI, GC9790 Plus). The temperature ranged from 100 to 300 °C. The NO conversion, CB conversion, N_2_ selectivity, and the yield of CO_2_ are calculated by the following equations:

 (1)

 (2)

 (3) (4)

where C*_NOx,inlet_*, C*_NH3,inlet_* and C*_C6H5Cl,inlet_* represent the inlet concentrations of NO_x_, NH_3_ and CB, respectively. And C*_N_*_Ox,outlet_, C*_NH3,outlet_*, C*_N2O,outlet_*, C*_C6H5Cl,outlet_* and C*_C_*_O2,outlet_ represent the outlet concentrations of NO*_x_*, NH_3_, N_2_O, CB and CO_2_, respectively.

The apparent activation energies (E_a_) for NH_3_-SCR and CBCO under individual and coupled reaction conditions were determined using the Arrhenius equation. The kinetic measurements were performed using 50mg (20mg) of catalyst. The individual NH_3_-SCR feed consisted of 500 ppm NO, 500 ppm NH_3_, and 5 vol.% O_2_. The individual CBCO feed consisted of 100 ppm CB and 5 vol.% O_2_. Under coupled conditions, the feed consisted of 100 ppm CB (450ppm), 500 ppm NO, 500 ppm NH_3_, and 5 vol.% O_2_. Ar was used as the balance gas. During each kinetic series, the inlet gas composition and total flow rate were maintained constant, and only the reaction temperature was varied.

The temperature range was selected independently for each target pollutant and reaction mode to maintain the conversion of the target pollutant below 20%. For the NO kinetic analysis, steady-state data were collected over 90-110 °C under individual NH_3_-SCR conditions and 100-130 °C under coupled conditions. For the CB kinetic analysis, steady-state data were collected over 155-175 °C under individual CBCO conditions and 190-225 °C under coupled conditions. Only data points at which the conversion of the target pollutant was below 20% were used for the Arrhenius fitting.

During the coupled CBCO measurements conducted over 190-225 °C, CB conversion remained below 20%, whereas NO conversion ranged from 69.52% to 75.99%. Therefore, the low-conversion criterion was applied specifically to CB, while NH_3_-SCR proceeded simultaneously to a substantial extent. The conversion of the non-target pollutant was not used as a criterion for selecting the kinetic data.

The rate constant K was calculated from the inlet and outlet molar flow rates of the corresponding target pollutant and normalized by the catalyst mass. E_a_ was obtained from the slope of ln K versus 1/T using the following equations. Because the co-reactants may affect competitive adsorption, surface coverage, oxygen consumption, and the overall reaction network, the E_a_ values obtained under coupled conditions represent overall apparent activation energies rather than activation barriers for individual elementary steps.

 (5)

 (6)

In Eq. (6), R represents the ideal gas constant, T is the temperature, and A is the pre-exponential factor.

When the NO*_x_* or CB conversion is below 20 %, the reaction rate of the SCR and the CBCO process (R_mass_ and R_BET_) can be calculated using the following equations:

 (7)

 (8)

 (9)

In Eq. (7), N_A_ represents Avogadro's constant, while C_in_ and C_out_ correspond to the molar flow rates of NO*_x_* or CB at the inlet and outlet, respectively. W_catal_ represents the catalyst mass. Additionally, R_BET_ in Eq. (8) refers to the Brunauer-Emmett-Teller (BET) specific surface area. where Mn is the XPS-derived Mn/(Mn + Ce + O) atomic fraction after excluding adventitious carbon.

## 1.10 MES (modulation excitation spectroscopy) experiments

In Fourier transform infrared (FTIR) spectroscopy experiments were conducted on an FTIR spectrometer (Thermo Fisher Scientific, Nicolet IS50) equipped with a mercury cadmium telluride detector and a Harrick reaction cell. For the concentration modulation excitation experiments, the solenoid valves were used to automatically switch between gases. The pulse sequence according to Figure 4e and 4f (NO+NH_3_+O_2_/CB+NH_3_+O_2_ modulation: 2000 ppm NO/Ar vs. 2000 ppm CB/Ar, constant 5 % O_2_/Ar and 2000ppm NH_3_/Ar) was introduced into the reaction cell at 200 °C. The set of time-resolved spectra obtained from the modulation experiments was converted into MES spectra using phase sensitive detection (PSD):

 (1)

where *I*(t) is the set of time-resolved data, ω the stimulation frequency, k the demodulation index (k = 1 is the fundamental harmonic and was used in this work), T the modulation period, and ϕ^PSD^ the phase angle. Python was used to process the time-resolved data using PSD. The modulation period (T = 240 s) is defined as the time required to conclude one full sequence. A single modulation period typically consisted of 240 consecutive time resolved FTIR spectra, identical modulation sequences were applied and consisted of 12 consecutives. The FTIR spectra were recorded as 8 scans at a resolution of 4cm^-1^.

## 1.11 DFT calculations

All density functional theory (DFT) calculations were performed using the Vienna Ab initio Simulation Package (VASP). Spin polarization was considered throughout. The generalized gradient approximation (GGA) with the Perdew-Burke-Ernzerhof (PBE) functional was adopted to describe the exchange-correlation energies. The projector augmented wave (PAW) method was used to describe the ionic cores, and valence electrons were treated with a plane wave basis set at a kinetic energy cutoff of 450 eV. Partial occupancies of the Kohn-Sham orbitals were allowed using the Gaussian smearing method with a width of 0.05 eV. The electronic energy was considered self-consistent when the energy change was smaller than 10^−5^ eV, and the convergence criterion for geometry relaxation was set to 0.03 eV∙Å^−1^. The Brillouin zones were sampled using 2 × 2 × 1 Monkhorst-Pack k-points for surface structures. To account for the strongly correlated nature of the 3d (Mn) and 4f (Ce) electrons, the DFT+U method was applied with the effective Hubbard U parameters of Ueff = 4.0 eV for Mn 3d and Ueff = 5.0 eV for Ce 4f, respectively, following well-established literature values for mixed MnO*_x_*-CeO_2_ systems. Spin-polarized calculations were performed throughout. For the magnetic configurations, both ferromagnetic (FM) and antiferromagnetic (AFM) couplings of the Mn ions were tested, and the AFM ground state was adopted as it yielded the lower total energy. Initial magnetic moments were set to 5 μB for Mn ions. Grimme’s DFT-D3 semiempirical dispersion correction was included to properly describe the van der Waals interactions between the adsorbed molecules and the oxide surfaces.

Surface slab models were constructed based on the CeO_2_ (111) facet (3 or 4 atomic layers) with a MnO*_x_* overlayer (1–2 ML) to represent the R-MnCe surface, and vice versa for N-MnCe. A vacuum layer of 15 Å was introduced to avoid spurious interactions between periodic images. For oxygen vacancy simulations, one lattice oxygen atom coordinated to Mn at the interface was removed, and the resulting structure was fully relaxed. For transition-state searching, the climbing-image nudged elastic band (CI-NEB) method was employed to identify the transition states of the oxygen activation and vacancy-replenishment pathways. Eight interpolated images were placed between the initial and final states, and the force convergence threshold was set to 0.05 eV Å^−1^. Vibrational frequency analysis was performed for the optimized transition states, confirming the presence of one imaginary frequency corresponding to bond cleavage/formation along the reaction coordinate, while the stable intermediates showed no imaginary frequencies. Zero-point energy, enthalpy, and entropy corrections were included to convert electronic energies into Gibbs free energies at 298.15 K for kinetic-barrier evaluation.

The adsorption energy () was calculated as:

 (4)

Where E_total_ is the total energy of the substrate with the adsorbate, E_substrate_ is the energy of the clean substrate, and E_adsorbate_ is the energy of the isolated adsorbate molecule.

The Gibbs free energy change (ΔG) for each elementary reaction step was calculated as:

(5)

Where ΔE is the difference in total energy, ΔE_ZPE_ is the difference in zero-point energy, ΔS is the change in entropy, and T is the temperature. The d-band center (εd) was calculated from the projected density of states (PDOS).

## 1.12 Electron paramagnetic resonance (EPR) measurements

Continuous-wave X-band EPR spectra were recorded using a JEOL JES X320 spectrometer at 77 K and 298 K. For each measurement, 20 mg of the powder sample was directly loaded into an EPR sample tube. The magnetic field was swept from 290 to 370 mT, with a center field of 330 mT and a sweep time of 30 s. The microwave frequency and microwave power were 9.230792 GHz and 0.998 mW, respectively. The modulation frequency was 100 kHz, and the modulation amplitude was 0.1 mT. The first-harmonic spectra were acquired using a time constant of 0.1 s and one accumulation. The spectra were interpreted qualitatively, and no spin quantification or determination of the relative Mn(II) contents was performed based on the signal amplitudes.

## 1.13 Kelvin probe force microscopy measurements

Kelvin probe force microscopy (KPFM) measurements were performed using a Dimension Icon atomic force microscope (Bruker, Germany) equipped with a conductive probe under ambient conditions at room temperature. Freshly cleaved highly oriented pyrolytic graphite (HOPG) with a reference surface potential of 4.60 eV was used to calibrate the probe. HOPG, N-MnCe, and R-MnCe were measured using the same probe and identical acquisition parameters.

Because the electrical bias was applied to the probe, the measured contact potential difference was defined as:

 (6)

where and are the surface potential of the probe and sample, respectively, and e is the elementary charge. The probe surface potential was first calibrated using HOPG:

 (7)

where The sample surface potential was subsequently calculated as:

 (8)

The calibrated surface potential of N-MnCe and R-MnCe were 4.731 and 4.583 eV, respectively. The 0.148 eV decrease after VROT indicates that the reconstructed Mn-enriched surface has a lower work function, consistent with its enhanced electron-donating tendency.

# Supplementary results and discussion

# 2. Catalyst characterizations

## 2.1 XRD patterns of CeO_2_, N-MnCe and R-MnCe catalyst standard card comparison diagram

**Figure S1** XRD patterns of CeO_2_, N-MnCe and R-MnCe catalyst standard card comparison diagram.

## 2.2 XRD patterns of N-MnCe and R-MnCe catalyst standard card comparison diagram

**Figure S2** XRD patterns of N-MnCe and R-MnCe catalyst standard card comparison diagram.

## 2.3 Whole-pattern XRD fitting and semiquantitative relative crystalline-phase fractions of N-MnCe and R-MnCe

**Figure S3** Whole-pattern XRD fitting and semiquantitative relative crystalline-phase fractions of N-MnCe and R-MnCe. The fitted fractions represent XRD-detectable crystalline contributions and do not include amorphous or poorly crystalline species.

## 2.4 HAADF images of N-MnCe catalyst

**Figure S4** HAADF image of N-MnCe catalyst, with illustration of selected area diffraction pattern.

## 2.5 HAADF image of R-MnCe catalyst

**Figure S5** HAADF image of R-MnCe catalyst, with illustration of selected area diffraction pattern.

## 2.6 XPS spectra of N-MnCe and R-MnCe catalyst

**Figure S6** XPS spectra of N-MnCe and R-MnCe catalyst.

## 2.7 XPS supplementary spectra of N-MnCe and R-MnCe catalyst

**Figure S7** XPS supplementary spectra of N-MnCe and R-MnCe catalyst.

## 2.8 In situ DRIFTS spectra of N-MnCe the sequential vacuum-O_2_ treatment

**Figure S8** *In situ* DRIFTS spectra of N-MnCe the sequential vacuum-O_2_ treatment.

## 2.9 Wavelet transforms for the k^3^-weighted Mn K-edge EXAFS signals of MnO_2_

**Figure S9** Wavelet transforms for the k^3^-weighted Mn K-edge EXAFS signals of MnO_2_.

## 2.10 In situ DRIFTS spectrum of the reaction between NO+NH_3_ and preadsorbed CB over N-MnCe

**Figure S10** *In situ* DRIFTS spectrum of the reaction between NO+NH_3_ and preadsorbed CB over N-MnCe at 200°C.

## 2.11 In situ DRIFTS spectrum of the reaction between NO+NH3 and preadsorbed CB over R-MnCe

**Figure S11** *In situ* DRIFTS spectrum of the reaction between NO+NH_3_ and preadsorbed CB over R-MnCe.

## 2.12 The structural model of (a) N-MnCe and (b) R-MnCe for DFT calculations.Ce,Yellow-green; Mn, purple; O, red

**Figure S12** The structural model of (a) N-MnCe and (b) R-MnCe for DFT calculations. Ce,Yellow-green; Mn, purple; O, red. The atomic structures represent idealized local Ce-rich and Mn-rich surface configurations used to evaluate the effects of Mn surface enrichment.

## 2.13 Density of States and d band center of (a) N-MnCe and (b) R-MnCe

**Figure S13** Density of States and d band center of (a) N-MnCe and (b) R-MnCe.

## 2.14 Adsorption and desorption curve of N-MnCe

**Figure S14** Adsorption and desorption curve of N-MnCe.

## 2.15 Adsorption and desorption curve of R-MnCe

**Figure S15** Adsorption and desorption curve of R-MnCe.

## 2.16 EPR spectra of R-MnCe after the coupled reaction at 200 °C for 3 h under O_2_-containing (5 vol.% O_2_) and O_2_-free conditions.

**Figure S16** EPR spectra of R-MnCe in 298K after the coupled reaction at 200 °C for 3 h under O_2_-containing (5 vol.% O_2_) and O_2_-free conditions.

## 2.17 EPR spectra of N-MnCe and R-MnCe catalyst at 77K

**Figure S17** EPR spectra of N-MnCe and R-MnCe catalyst at 77K.

## 2.18 Deconvoluted O 1s XPS spectra of R-MnCe after the coupled reaction at 200 °C for 3 h under O_2_-containing (5 vol.% O_2_) and O_2_-free conditions.

**Figure S18** Deconvoluted O 1s XPS spectra of R-MnCe after the coupled reaction at 200 °C for 3 h under O_2_-containing (5 vol.% O_2_) and O_2_-free conditions.

## 2.19 XRD patterns of Mo/Ce and W/Ce catalysts before and after the sequential vacuum-O_2_ treatment

**Figure S19** XRD patterns of Mo/Ce and W/Ce catalysts before and after the sequential vacuum-O_2_ treatment.

## 2.20 H_2_-TPR profiles of Mo/Ce and W/Ce catalysts before and after the sequential vacuum-O_2_ treatment.

**Figure S20** H_2_-TPR profiles of Mo/Ce and W/Ce catalysts before and after the sequential vacuum-O_2_ treatment.

## 2.21 XRD patterns of Mn_x_Ce_x_ catalysts before and after the sequential vacuum-O_2_ treatment.

**Figure S21** XRD patterns of Mn*_x_*Ce*_x_* catalysts before and after the sequential vacuum-O_2_ treatment.

## 2.22 H_2_-TPR profiles of Mn_x_Ce_x_ catalysts before and after the sequential vacuum-O_2_ treatment

**Figure S22** H_2_-TPR profiles of Mn*_x_*Ce*_x_* catalysts before and after the sequential vacuum-O_2_ treatment.

## 2.23 XRD patterns of R-MnCe and N-MnCe and different treatment processes catalysts

**Figure S23** XRD patterns of R-MnCe and N-MnCe and different treatment processes catalysts.

## 2.24 H_2_-TPR profiles of R-MnCe and N-MnCe and different treatment processes catalysts

**Figure S24** H_2_-TPR profiles of R-MnCe and N-MnCe and different treatment processes catalysts.

## 2.25 XRD patterns of R-MnCe and MnCe-PM and MnCe-Imp catalysts

**Figure S25** XRD patterns of R-MnCe and MnCe-PM and MnCe-Imp catalysts.

## 2.26 H_2_-TPR profiles of R-MnCe and MnCe-PM and MnCe-Imp catalysts

**Figure S26** H_2_-TPR profiles of R-MnCe and MnCe-PM and MnCe-Imp catalysts.

## 2.27 Physical properties of N-MnCe and R-MnCe

**Table S1** Physical properties of N-MnCe , R-MnCe and other, atomic content was measured by XPS, BET and average pore size were measured by chemical adsorption instrument.

| sample | C  （At%） | Ce  (At%) | Mn  (At%) | O  (At%) | Mn/Ce  (At%) | S_BET_  (m^2^/g) | Average pore diameter  (nm) |
| --- | --- | --- | --- | --- | --- | --- | --- |
| N-MnCe | 53.26 | 5.13 | 5.17 | 36.44 | 1.01 | 83.9081 | 9.9797 |
| R-MnCe | 48.07 | 4.77 | 6.17 | 40.98 | 1.29 | 83.7863 | 10.0981 |
| R-MnCe-2 | 28.52 | 8.54 | 10.42 | 52.52 | 1.22 | - | - |
| R-MnCe-3 | 56.84 | 3.85 | 4.84 | 34.47 | 1.26 | - | - |
| MnCe-V | 28.78 | 9.30 | 10.48 | 51.44 | 1.12 | - | - |
| MnCe-O | 28.13 | 9.09 | 10.81 | 51.97 | 1.18 | - | - |
| MnCe-VAr | 28.08 | 9.50 | 10.54 | 51.88 | 1.10 | - | - |
| MnCe-Imp | 30.58 | 7.56 | 10.95 | 50.9 | 1.45 | - | - |
| MnCe-PM | 32.70 | 8.06 | 8.46 | 50.77 | 1.05 | - | - |
| Mn_0.5_Ce_1_ | 28.26 | 12.14 | 6.47 | 53.14 | 0.53 | - | - |
| Mn_0.5_Ce_1_-R | 29.23 | 11.5 | 6.56 | 52.65 | 0.57 | - | - |
| Mn_0.6_Ce_1_ | 27.56 | 12.40 | 6.59 | 53.44 | 0.53 | - | - |
| Mn_0.6_Ce_1_-R | 39.99 | 8.44 | 4.83 | 46.74 | 0.57 | - | - |
| Mn_2_Ce_1_ | 27.72 | 6.55 | 13.39 | 52.33 | 2.04 | - | - |
| Mn_2_Ce_1_-R | 26.16 | 6.45 | 14.47 | 52.93 | 2.24 | - | - |

**Table S2** Physical properties of WO*_x_*/Ce, Mo/Ce , atomic content was measured by XPS. The theoretical values are all 2.23 wt.%.

| sample | C  （At%） | Ce  (At%) | W  (At%) | Mo  (At%) | O  (At%) | W/(W+Ce+O)  (At%) | Mo /( Mo +Ce+O)  (At%) |
| --- | --- | --- | --- | --- | --- | --- | --- |
| WOx/Ce | 29.86 | 15.86 | 1.58 | - | 52.70 | 2.25 | - |
| WOx/Ce-R | 30.31 | 15.81 | 1.77 | - | 52.31 | 2.54 | - |
| Mo/Ce | 28.23 | 16.45 | - | 2.00 | 53.31 | - | 2.78 |
| Mo/Ce-R | 28.59 | 16.00 | - | 2.14 | 53.46 | - | 3.00 |

# 3. Catalytic Performance

## 3.1 SCR performance of N-MnCe catalyst modified at different temperatures for removing NO_x_ and CB

**Figure S27** SCR performance of N-MnCe catalyst modified at different temperatures for removing NO*_x_* and CB. Error bars represent the standard deviation from three independent measurements (n = 3).

## 3.2 CB oxidation performance of N-MnCe catalyst modified at different temperatures for removing NO_x_ and CB

**Figure S28** CB oxidation performance of N-MnCe catalyst modified at different temperatures for removing NO*_x_* and CB. Error bars represent the standard deviation from three independent measurements (n = 3).

## 3.3 SCR performance of N-MnCe catalysts modified with different vacuum times for removing NO_x_ and CB

**Figure S29** SCR performance of N-MnCe catalysts modified with different vacuum times for removing NO*_x_* and CB. Error bars represent the standard deviation from three independent measurements (n = 3).

## 3.4 CB oxidation performance of N-MnCe catalysts modified with different vacuum times for removing NO_x_ and CB

**Figure S30** CB oxidation performance of N-MnCe catalysts modified with different vacuum times for removing NO*_x_* and CB. Error bars represent the standard deviation from three independent measurements (n = 3).

## 3.5 SCR performance of the catalytic performance of N-MnCe and R-MnCe catalysts in the coupled removal of NO_x_ and CB

**Figure S31** SCR performance of N-MnCe and R-MnCe catalysts in the coupled removal of NO*_x_* and CB. Error bars represent the standard deviation from three independent measurements (n = 3).

## 3.6 CB oxidation performance of N-MnCe and R-MnCe catalysts in the coupled removal of NO_x_ and CB

**Figure S32** CB oxidation performance of N-MnCe and R-MnCe catalysts in the coupled removal of NO*_x_* and CB. Error bars represent the standard deviation from three independent measurements (n = 3).

## 3.7 N_2_ selectivity of N-MnCe and R-MnCe catalysts in the coupled removal of NO_x_ and CB

**Figure S33** N_2_ selectivity of N-MnCe and R-MnCe catalysts in the coupled removal of NO*_x_* and CB. Error bars represent the standard deviation from three independent measurements (n = 3).

## 3.8 CO_2_ yield of N-MnCe and R-MnCe catalysts in the coupled removal of NO_x_ and CB

**Figure S34** CO_2_ yield of N-MnCe and R-MnCe catalysts in the coupled removal of NO*_x_* and CB. Error bars represent the standard deviation from three independent measurements (n = 3).

## 3.9 SCR performance of Mo/Ce and W/Ce catalysts before and after the sequential vacuum-O_2_ treatment in the coupled removal of NO_x_ and CB

**Figure S35** SCR performance of Mo/Ce and W/Ce catalysts before and after the sequential vacuum-O_2_ treatment in the coupled removal of NO*_x_* and CB. Error bars represent the standard deviation from three independent measurements (n = 3).

## 3.10 CB oxidation performance of Mo/Ce and W/Ce catalysts before and after the sequential vacuum-O_2_ treatment in the coupled removal of NO_x_ and CB

**Figure S36** CB oxidation performance of Mo/Ce and W/Ce catalysts before and after the sequential vacuum-O_2_ treatment in the coupled removal of NO*_x_* and CB. Error bars represent the standard deviation from three independent measurements (n = 3).

## 3.11 SCR performance of Mn_x_Ce_x_ catalysts before and after the sequential vacuum-O_2_ treatment in the coupled removal of NO_x_ and CB

**Figure S37** SCR performance of Mn*_x_*Ce*_x_* catalysts before and after the sequential vacuum-O_2_ treatment in the coupled removal of NO*_x_* and CB. Error bars represent the standard deviation from three independent measurements (n = 3).

## 3.12 CB oxidation performance of Mn_x_Ce_x_ catalysts before and after the sequential vacuum-O_2_ treatment in the coupled removal of NO_x_ and CB

**Figure S38** CB oxidation performance of Mn*_x_*Ce*_x_* catalysts before and after the sequential vacuum-O_2_ treatment in the coupled removal of NO*_x_* and CB at 200°C. Error bars represent the standard deviation from three independent measurements (n = 3).

## 3.13 CB oxidation performance of R-MnCe and different treatment processes catalysts in the coupled removal of NO_x_ and CB at 200 °C

**Figure S39** CB oxidation performance of R-MnCe and different treatment processes catalysts in the coupled removal of NO*_x_* and CB at 200 °C. Error bars represent the standard deviation from three independent measurements (n = 3).

## 3.14 CB oxidation performance of R-MnCe and MnCe-PM and MnCe-Imp catalysts in the coupled removal of NO_x_ and CB at 200 °C

**Figure S40** CB oxidation performance of R-MnCe and MnCe-PM and MnCe-Imp catalysts in the coupled removal of NO*_x_* and CB at 200 °C. Error bars represent the standard deviation from three independent measurements (n = 3).

## 3.15 Long-term stability of R-MnCe under coupled NO_x_-chlorobenzene removal conditions at 200 °C.

**Figure S41** Long-term stability of R-MnCe under coupled NO*_x_*-chlorobenzene removal conditions at 200 °C.

## 3.16 H_2_O and SO_2_ tolerance of R-MnCe under coupled NO_x_-chlorobenzene removal conditions at 250 °C.

**Figure S42** H_2_O and SO_2_ tolerance of R-MnCe under coupled NO*_x_*-chlorobenzene removal conditions at 250 °C.

## 3.17 Comparison of R_mass,NO_ with other catalysts at 150 °C and R_mass,CB_ with other catalysts at 200 °C.

**Table S3** Comparison of R_mass,NO_ with other catalysts at 150 °C and R_mass,CB_ with other catalysts at 200 °C

| **Catalyst** | **NO conversion**  **（%）** | **CB conversion**  **（%）** | **NO**  **Concentration**  **(ppm)** | **CB**  **Concentration**  **(ppm)** | **Catalyst**  **(g)** | **GHSV**  **（h^-1^）** | **R_mass,NO_**  **(10^16^ g^-1^s^-1^)** | **R_mass,CB_**  **(10^16^ g^-1^s^-1^)** | **Ref.** |
| --- | --- | --- | --- | --- | --- | --- | --- | --- | --- |
| N-MnCe | 5.164(0.02) | 2.89(0.05) | 500 | 450 | 0.02/0.05 | 50000 | 13.63 | 6.86 | This work |
| R-MnCe | 17.61(0.02) | 12.22(0.05) | 500 | 450 | 0.02/0.05 | 50000 | 46.48 | 28.98 | This work |
| 12HPW-CeO_2_ | 95 | 39 | 500 | 100 | 0.2 | 50000 | 14.52 | 2.06 | [1] |
| MnO_2_-CePO_4_/TiO_2_ | 88 | 11 | 500 | 100 | 0.1 | 50000 | 15.31 | 0.58 | [2] |
| Co-SM | 100 | 41 | 500 | 100 | 0.2 | 50000 | 23.75 | 2.16 | [3] |
| 8V/TiO_2_ | 89 | 10 | 600 | 100 | 0.1 | 40000 | 2.28 | 0.42 | [4] |
| Al/Ce | 90 | 18 | 500 | 500 | 0.1 | 40000 | 12.67 | 3.80 | [5] |
| Ta/Ce | 85 | 15 | 500 | 500 | 0.1 | 40000 | 7.60 | 3.17 | [5] |
| P/MnO_2_-CeO_2_ | 93 | 28 | 500 | 100 | 0.1 | 60000 | 0.32 | 1.77 | [6] |
| SbPdV/TiO_2_ | 90 | 9 | 600 | 600 | 0.1 | 30000 | 7.60 | 1.71 | [7] |
| Co-MnO*_x_* | 93 | 22 | 500 | 100 | 0.2 | 60000 | 31.67 | 3.17 | [8] |

**Table S4** Apparent reaction rates normalized by catalyst mass, BET surface area, and surface Mn content.

| Condition | Reaction | Sample | R_mass_ (g^-1^s^-1^) | R_BET_ (m^-2^s^-1^) | R_app,Mn_ (m^-2^s^-1^) |
| --- | --- | --- | --- | --- | --- |
| Individual | NO | N-MnCe | 1.2044E+16 | 1.43538E+14 | 1.29769E+15 |
| Individual | NO | R-MnCe | 2.4088E+16 | 2.87493E+14 | 2.41916E+15 |
| Individual | CB | N-MnCe | 2.33776E+16 | 2.7861E+14 | 2.51885E+15 |
| Individual | CB | R-MnCe | 3.74052E+16 | 4.46436E+14 | 3.75661E+15 |
| Coupled | NO | N-MnCe | 1.36291E+17 | 1.62428E+15 | 1.46848E+16 |
| Coupled | NO | R-MnCe | 4.64771E+17 | 5.5471E+15 | 4.66771E+16 |
| Coupled | CB | N-MnCe | 6.86468E+16 | 8.18119E+14 | 7.39643E+15 |
| Coupled | CB | R-MnCe | 2.89789E+17 | 3.45867E+15 | 2.91036E+16 |

# 4. EPR Measurements and Qualitative Analysis of Mn Species

As shown in Figure 2i, both N-MnCe and R-MnCe exhibit a weak six-line hyperfine feature around g≈1.98. This feature is characteristic of high-spin Mn(II) (*S* = 5/2) and arises from hyperfine coupling between the electron spin and the ^55^Mn nucleus (*I* = 5/2). The spectra therefore provide qualitative evidence for the presence of Mn(II) species in both samples, consistent with the Mn(II) component identified by Mn 2p XPS.

The EPR spectra were neither double-integrated nor calibrated using a quantitative EPR protocol. Consequently, the signal amplitudes were not used to compare the Mn(II) contents of N-MnCe and R-MnCe because they may also be influenced by linewidth, powder packing, resonator quality factor, and sample electrical conductivity. The observed feature does not establish the local coordination environment or catalytic role of the Mn(II) species. Moreover, the measurements conducted at 77 K do not exclude the coexistence of Mn(III) or Mn(IV) species, whose EPR responses may be weak, broad, unresolved, or unobservable under the present conditions.

# 5. Further Evaluation of the Sequential Vacuum–O_2_ Treatment

To examine the feasibility of the sequential vacuum–O_2_ treatment in other ceria-based mixed oxides, Mo/Ce and W/Ce catalysts were subjected to the same procedure. For Mo/Ce, NO conversion increased from approximately 1% to 13% at 100 °C and from 16% to 29% at 150 °C, while CB conversion increased from 22% to 34% at 350 °C after treatment (Figures S35 and S36). XPS showed that the surface Mo/(Mo + Ce + O) fraction increased from 2.78% to 3.00%. The fluorite CeO_2_ structure was retained without detectable crystalline MoO*_x_* phases (Figure S19). Meanwhile, the main H_2_-TPR peak shifted from 435 to 465 °C with increased signal intensity, indicating that the treatment altered the distribution and reduction behavior of the Mo-containing species (Figure S20).

For W/Ce, the treatment increased CB conversion from approximately 30% to 43% at 150 °C, from 46% to 57% at 200 °C, and from 69% to 83% at 300 °C, while NO conversion was slightly improved or maintained over the investigated temperature range (Figures S35 and S36). The surface W/(W + Ce + O) fraction increased from 2.25% to 2.54%, while the fluorite CeO_2_ structure remained preserved without detectable crystalline WO*_x_* phases (Figure S19). The main H_2_-TPR peak shifted from 483 to 501 °C, accompanied by the appearance of an additional reduction feature at approximately 363 °C. This low-temperature feature is consistent with the formation of more readily reducible W-containing surface or interfacial species after treatment (Figure S20).

The influence of the sequential vacuum–O_2_ treatment was further examined using Mn-Ce oxides with different nominal Mn/Ce ratios. At 200 °C, CB conversion increased from approximately 21.5% to 47.2% for Mn_0.5_Ce_1_, from 34.4% to 58.7% for Mn_0.6_Ce_1_, and from 34.2% to 88.3% for Mn_2_Ce_1_ after treatment (Figure S38). For Mn_0.5_Ce_1_, NO conversion at 100 °C increased from approximately 24% to 90%, while high NO conversions were maintained for the other compositions (Figure S37). XPS showed corresponding increases in the surface Mn/Ce ratio from 0.53 to 0.57, 0.53 to 0.57, and 2.04 to 2.24, respectively (Table S1), demonstrating treatment-induced near-surface Mn enrichment across the investigated compositions. The XRD and H_2_-TPR results further showed changes in crystallinity and reduction characteristics after treatment (Figures S21 and S22). In particular, the high-temperature reduction features shifted from 382 to 370 °C for Mn_2_Ce_1_ and from 397 to 390 °C for Mn_0.6_Ce_1_, while the low-temperature feature of Mn_0.5_Ce_1_ shifted from 220 to 217 °C. These results show that the sequential vacuum–O_2_ treatment can regulate the near-surface Mn distribution and redox properties of Mn-Ce oxides with different compositions, accompanied by improved coupled catalytic performance.

The respective contributions of the vacuum and O_2_ steps were examined using MnCe-V, MnCe-O, and MnCe-VAr. At 200 °C, CB conversions were approximately 91.5%, 49.9%, and 89.7%, respectively, compared with nearly 100% over R-MnCe (Figure S39). Their surface Mn/Ce ratios were 1.12, 1.18, and 1.10, respectively, all lower than the average value of 1.26 ± 0.03 for R-MnCe (Table S1). Although each individual treatment produced more distinct diffraction peaks than N-MnCe, none reproduced the XRD profile of R-MnCe (Figure S23). Similarly, the control samples exhibited reduction features at approximately 223 and 362 °C, whereas R-MnCe displayed a characteristic multistep reduction profile at approximately 193, 291, and 362 °C (Figure S24). These results indicate that neither treatment step alone reproduces the near-surface composition, reduction behavior, and catalytic performance generated by the complete sequential vacuum–O_2_ treatment.

Conventional impregnation and physical mixing were also examined. At 200 °C, MnCe-Imp and MnCe-PM achieved approximately 38% and 20% CB conversion, respectively, substantially lower than the nearly complete conversion over R-MnCe (Figure S40). The surface Mn/Ce ratios were 1.45 for MnCe-Imp and 1.05 for MnCe-PM (Table S1). Notably, the higher surface Mn/Ce ratio of MnCe-Imp did not result in activity comparable to R-MnCe, indicating that surface Mn abundance alone cannot explain the catalytic enhancement. MnCe-Imp exhibited reduction peaks at approximately 257 and 346 °C, whereas MnCe-PM showed a dominant reduction peak at approximately 471 °C; neither reproduced the multistep reduction profile of R-MnCe (Figures S25 and S26). Their XRD patterns also contained sharp reflections associated with separated crystalline oxide phases, distinct from the reconstructed structure of R-MnCe.

Overall, these results demonstrate that the properties of R-MnCe cannot be reproduced by an individual treatment step, conventional impregnation, or physical mixing. The complete sequential vacuum–O_2_ treatment promotes near-surface metal redistribution and modifies the associated interfacial redox behavior. The results obtained for different Mn-Ce compositions and the preliminary Mo/Ce and W/Ce systems further support the feasibility and potential extension of this post-synthetic surface-reconstruction approach.

# Supplementary References

[1]	H. Yang, A. Chen, F. Wang, T. Lan, J. Zhang, X. Hu, Y. Shen, D. Cheng, D. Zhang, “Phosphotungstic Acid as a Dechlorination Agent Collaborates with CeO_2_ for Synergistic Catalytic Elimination of NO*_x_* and Chlorobenzene” *Environ. Sci. Technol.* **2024**, *58*, 7672–7682.

[2]	C. Zhang, J. Zhang, Y. Shen, J. He, W. Qu, J. Deng, L. Han, A. Chen, D. Zhang, “Synergistic Catalytic Elimination of NO*_x_* and Chlorinated Organics: Cooperation of Acid Sites” *Environ. Sci. Technol.* **2022**, *56*, 3719–3728.

[3]	F. Wang, A. Chen, T. Lan, X. Chen, M. Wang, X. Hu, P. Wang, D. Cheng, D. Zhang, “Synergistic catalytic removal of NOx and chlorinated organics through the cooperation of different active sites” *Journal of Hazardous Materials* **2024**, *468*, 133722.

[4]	S. Zhai, Y. Su, X. Weng, R. Li, H. Wang, Z. Wu, “Synergistic Elimination of NO*_x_* and Chlorinated Organics over VO*_x_*/TiO_2_ Catalysts: A Combined Experimental and DFT Study for Exploring Vanadate Domain Effect” *Environ. Sci. Technol.* **2021**, *55*, 12862–12870.

[5]	L. Wei, Y. Liu, H. Dai, S. Cui, C. Wang, H.-C. Hsi, E. Duan, Y. Peng, J. Deng, “Electronic structure tailoring of Al^3+^- and Ta^5+^-doped CeO_2_ for the synergistic removal of NO and chlorinated organics” *Applied Catalysis B: Environmental* **2022**, *304*, 120939.

[6]	X. Zhu, X. Yuan, Z. Song, Y. Peng, J. Li, “A dual-balance strategy via phosphate modification on MnO_2_-CeO_2_ for NO*_x_* and chlorobenzene synergistic catalytic control” *Applied Catalysis B: Environmental* **2024**, *342*, 123364.

[7]	G. Li, S. Ding, X. Hou, K. Shen, S. Zhang, Y. Zhang, “Unlocking low-temperature and anti-SO_2_ poisoning performance of bimetallic PdV/TiO_2_ catalyst for chlorobenzene/NO*_x_* catalytic removal by antimony modification design” *Chemical Engineering Journal* **2023**, *457*, 141210.

[8]	D. Kang, Q. Shi, C. Zhang, P. Zhao, H. Lyu, M. Yang, Y. Bian, B. Shen, “Synergistic removal of NO*_x_* and CB by Co-MnO*_x_* catalysts in a low-temperature window” *Chemical Engineering Journal* **2023**, *476*, 146369.

[9] B. Ravel, M. Newville, “ATHENA, ARTEMIS, HEPHAESTUS: data analysis for X-ray absorption spectroscopy using IFEFFIT” Journal of Synchrotron Radiation 2005, 12, 537–541.
